# The Association Between Serum HMGB2 Levels and Abdominal Aortic Aneurysm in Males: Insights Into the HMGB2–TREM Pathway

**DOI:** 10.31083/RCM33511

**Published:** 2025-07-18

**Authors:** Liting Pan, Junji Chen, Yanjun Sun, Fang Wang

**Affiliations:** ^1^Department of Cardiology, Jiading Branch of Shanghai General Hospital, Shanghai Jiao Tong University School of Medicine, 800 Huangjiahuayuan Road, 201803 Shanghai, China; ^2^Department of Cardiovascular Surgery, Ruijin Hospital, Shanghai Jiao Tong University School of Medicine, 197 Ruijin Er Road, 200025 Shanghai, China; ^3^Department of Cardiovascular Medicine, Ruijin Hospital, Shanghai Jiao Tong University School of Medicine, 197 Ruijin Er Road, 200025 Shanghai, China; ^4^Department of Cardiovascular Medicine, Ruijin-Hainan Hospital, Shanghai Jiao Tong University School of Medicine, 41 Kangxiang Road, 571473 Qionghai, Hainan, China

**Keywords:** abdominal aortic aneurysm, HMGB1, HMGB2, sTREM-1, sTREM-2

## Abstract

**Background:**

Abdominal aortic aneurysm (AAA) is a major public health challenge and presents high mortality due to diagnostic and therapeutic difficulties. This study investigated the role of high-mobility group box2 (HMGB2) and the HMGB2-triggering receptor expressed on the myeloid cell (TREM) pathway in male AAA patients. The goal was to evaluate HMGB2 as a novel biomarker and to elucidate its contribution to the pathogenesis of AAA. Our findings offer new insights into AAA biology and highlight the potential application of HMGB2 for early detection and therapeutic targeting.

**Methods:**

This retrospective case–control study included 36 male AAA patients and 41 male controls with balanced baseline characteristics. HMGB1, HMGB2, soluble TREM-1 (sTREM-1), and sTREM-2 serum levels were measured by enzyme-linked immunosorbent assay (ELISA). The association between HMGB2 and AAA was analyzed using multivariate logistic regression, while the diagnostic performance of HMGB2 was assessed using receiver operating characteristic (ROC) curves.

**Results:**

Elevated HMGB2 and HMGB1 levels were associated with higher risks of AAA (HMGB2: OR: 1.158, 95% CI: 1.011–1.325; *p *< 0.05; HMGB1: OR: 1.275, 95% CI: 1.048–1.551; *p *< 0.05) and aneurysm rupture (HMGB2: OR: 1.117, 95% CI: 1.005–1.241; *p *< 0.05; HMGB1: OR: 1.212, 95% CI: 1.003–1.465; *p *< 0.05). Meanwhile, sTREM-1 exhibited a negative correlation with AAA (OR: 0.991, 95% CI: 0.985–0.997; *p *< 0.01). The odds ratios of the fourth quartile HMGB2 and HMGB1 levels for AAA were 6.925-fold and 8.621-fold higher, respectively, than the first quartile levels. The HMGB2 serum level was positively correlated with a larger AAA diameter, with the diameter increasing progressively as the HMGB2 level increased. The area under the ROC curve (AUC) for predicting AAA was 0.713 for HMGB2, 0.677 for HMGB1, and 0.665 for sTREM-1. HMGB1 and sTREM-1 both correlated with HMGB2. Each HMGB1 quartile group exhibited a significant increase as HMGB2 increased. Further, sTREM-1 significantly increased at low to moderate HMGB2 levels but decreased in the highest HMGB2 quartile.

**Conclusion:**

Elevated HMGB2 serum levels are independently associated with the incidence of AAA in males. HMGB2–TREM pathway disruption may play a critical role in AAA pathogenesis.

## 1. Introduction

Abdominal aortic aneurysm (AAA) is characterized by a permanent, localized dilation of the abdominal aorta that exceeds its normal diameter by 50%, or reaches a maximal diameter of 30 mm [1]. The majority of AAAs are located in the infrarenal aorta, proximal to the aortic bifurcation. The risk of AAA rupture escalates with increasing aortic diameter [2]. Non-syndromic AAA is a major cause of cardiovascular mortality due to the high risk of aortic rupture. The diagnosis of this condition is challenging because most aneurysms remain asymptomatic until rupture occurs [3, 4]. The incidence of AAA rupture in the American population between 2005 and 2012 was 7.29 per 100,000, accounting for 4%–5% of sudden death cases. Approximately 50% of patients who undergo AAA rupture reach hospital. The operative mortality rate is around 50%, although the exact figure is difficult to determine [4]. In 2017, AAA was responsible for more than 167,000 deaths globally, and 3 million disability-adjusted life years [5, 6]. In 2019, 35.12 million cases of AAA were reported world-wide among individuals aged 30–79 years (0.92%) [7]. Endovascular aneurysm repair (EVAR) and open surgery are the two surgical interventions for AAA repair [8]. While minimally invasive devices for EVAR have improved greatly, the durability of this treatment remains problematic, and the potential for rupture remains [9]. The development of novel therapies, including stem cell therapies, has faced significant challenges. Consequently, a strong imperative is to investigate the mechanisms underlying AAA formation and identify novel specific biomarkers or therapeutic approaches for diagnosing and treating AAA in its early stages.

Substantial evidence indicates the major contributors to AAA are chronic inflammation and dysregulation of the extracellular matrix (ECM), composed primarily of elastin and collagen proteins, and the loss of vascular smooth muscle cells (VSMCs) [10, 11]. The high-mobility group box (HMGB) family is comprised of four members (HMGB1-4) that play an important role in various inflammatory diseases and have the capacity to modulate innate immunity [12]. Research indicates that elevated HMGB1 levels are present in the aneurysmal tissue of human AAA and in murine experimental models of AAA [13]. The studies to date have predominantly focused on HMGB1, which drives pro-inflammatory signaling via receptors such as toll-like receptor (TLR) and advanced glycation end product (RAGE) [14, 15, 16]. Despite sharing >80% structural homology with HMGB1, HMGB2 exhibits distinct functional properties, including different expression patterns and interactions with immune pathways such as triggering receptor expressed on myeloid cells-1 (TREM-1) [17]. Previous clinical studies have demonstrated that elevated HMGB2 expression correlates with the severity of myocardial infarction (MI), major adverse cardiovascular events (MACEs) at one month, and in-stent restenosis [18, 19]. However, the role of HMGB2 in AAA requires further investigation. Wu *et al*. [20] reported increased HMGB2 levels in angiotensin-II-treated VSMCs, and in an angiotensin II-induced mouse model of AAA. Inhibition of HMGB2-regulated ferroptosis and inflammation in angiotensin-II-treated VSMCs may protect against AAA by inactivating nuclear factor-kappa B (NF-κβ) signaling. Despite these observations, there is a paucity of mechanistic evidence that directly connects HMGB2 to AAA development or rupture. TREM-1 is an immunoglobulin (Ig) superfamily member containing an Ig-like extracellular domain. It is closely associated with inflammatory reactions and various pathologies [21, 22]. Previous research has demonstrated that TREM-1 can exacerbate experimental AAA by modulating angiotensin II-induced monocyte trafficking and vascular wall inflammation [23]. Although the specific ligands for TREM-1 have yet to be definitively identified, several studies have suggested that HMGB1 could be a potential TREM-1 ligand involved in the inflammatory response [24, 25]. However, the interplay between HMGB2 and TREM-1, a key amplifier of inflammatory responses in AAA, has not yet been investigated.

The present study aims to address this gap in knowledge by investigating the specific contribution of HMGB2 to the pathogenesis of AAA, including the potential involvement of the HMGB2-TREM pathway. This should elucidate the potential value of HMGB2 for early detection and therapeutic targeting of AAA.

## 2. Materials and Methods

### 2.1 Study Participants

This retrospective case-control study included 77 consecutive male participants admitted to the cardiovascular surgery department of Ruijin Hospital from January 2019 to November 2021. All participants were screened for AAA.

The inclusion criteria were: (1) male adults who were clinically diagnosed with AAA; (2) the diameter of the aneurysm as determined by computed tomography (CT) examination was >45 mm [26]. To avoid confounding variables, patients with one or more of the following conditions were excluded from the study: acute heart failure, acute myocardial infarction, coronary heart disease, valvular heart disease, cardiomyopathy, sustained arrhythmias, concomitant stroke, renal or hepatic failure, acute/chronic infectious diseases, autoimmune diseases. A total of 36 patients with AAA exhibiting a diameter of >45 mm were classified into the AAA group, while 41 individuals who were screened and found to be free of arterial aneurysms served as the control group.

### 2.2 Definitions

The diagnostic criterion for AAA were the updated 2014 guidelines from the European Society of Cardiology (ESC), i.e., an abnormal local dilation of the infra-renal aorta at least 1.5-times greater than the normal aortic diameter at the level of the renal artery (minimum diameter about 30 mm) [27]. Smoking status was categorized as current (daily or at least monthly smoking), former (ceased smoking for at least one month), or never smoked [28]. Hypertension was identified as a systolic blood pressure ≥140 mmHg, diastolic blood pressure ≥90 mmHg, or the use of antihypertensive medication [29]. The criterion for diabetes were as follows: (1) fasting blood glucose (FBG) ≥7 mmol/L; (2) 2-h post-meal blood glucose ≥11.1 mmol/L; (3) glycosylated hemoglobin (HbA1c) ≥6.5%; (4) treatment with any oral hypoglycemic agent or insulin [30]. The criterion for dyslipidemia were as follows: (1) hypercholesterolemia: total cholesterol (TC) ≥5.2 mmol/L, low-density lipoprotein cholesterol (LDL-C) ≥3.4 mmol/L; (2) hypertriglyceridemia: triglycerides (TG) ≥1.7 mmol/L; (3) mixed hyperlipoproteinemia: TC ≥5.2 mmol/L and TG ≥1.7 mmol/L; (4) low high-density lipoprotein cholesterol (HDL-C): HDL-C <1.0 mmol/L; (5) hyperlipoproteinemia (a): lipoprotein(a) ≥300 mg/L; (6) treatment with any kind of lipid-lowering therapy [31]. Clinical data and characteristics of all participants were obtained from the medical record system, including age, vital signs on admission, medical history (including smoking status, hypertension, hyperlipidemia, and diabetes), resting echocardiographic parameters, and other clinical reports. The AAA and control groups demonstrated balanced baseline characteristics, including gender (all male), age, blood pressure, and heart rate on admission. All procedures were performed according to the principles of the Declaration of Helsinki and approved by the local Research and Ethics Committee of Ruijin Hainan Hospital (ethical approval number: 2023 (No.70)). Informed written consent was obtained from all participants.

### 2.3 Biochemical Measurements and Echocardiography

Peripheral venous blood samples were collected from all participants after overnight fasting. Standard laboratory techniques were used to measure creatinine, estimated glomerular filtration rate (eGFR), TG, TC, HDL-C, LDL-C, FBG, HbA1c, troponin I (TnI), N-terminal pro-brain natriuretic peptide (NT-pro BNP), and D-Dimer. These were performed using the HITACHI 912 Analyzer (Roche Diagnostics, Mannheim, Germany) in the Clinical Laboratory, Ruijin Hospital. Standard echocardiography (Vivid E95, GE Healthcare, Chicago, IL, USA) was performed within 72 h of admission, and left ventricular ejection fraction (LVEF) was calculated using the biplane modified Simpson’s method.

### 2.4 Measurement of Serum HMGB1, HMGB2, sTREM-1 and sTREM-2 Levels

Blood samples were transferred immediately into pyrogen-free tubes, centrifuged immediately at 1500 r/min for 15 min at 4 °C, and then stored in aliquots at –80 °C until analysis. Serum levels of soluble TREM-1 (sTREM-1) and sTREM-2 were determined with commercially available enzyme-linked immunosorbent assay (ELISA) kits according to standardized protocols (human TREM-1 duoset, DY1278B, R&D System, Minneapolis, MN, USA; human TREM-2 duoset, DY1828-05, R&D System, Minneapolis, MN, USA). Human HMGB1 matched antibody pair (H00003146-AP41) and human HMGB2 monoclonal antibody (H00003148-M03) were both purchased from Abnova Corporation (Taipei, Taiwan), while polyclonal HMGB2 antibody (H9789) was purchased from Sigma-Aldrich (St Louis, MO, USA). The serum levels of HMGB1 and HMGB2 were determined by sandwich ELISA as described previously by our laboratory [18, 19]. Briefly, for the evaluation of HMGB2, biotinylated monoclonal anti-HMGB2 antibody (H00003148-M03) was incubated in streptavidin-coated and blocked wells for 1 h and then washed. Serum samples or HMGB2 calibrator standards were diluted in 100 µL assay buffer containing 50 mM sodium phosphate (pH 7.4), 0.5% bovine serum albumin (BSA), 5 mM ethylenediaminetetraacetic acid (EDTA), and 0.001% aprotinin. This mixture was placed into the wells and incubated at room temperature for 1 h. After washing, the captured HMGB2 molecules were identified using a polyclonal HMGB2 antibody (H9789) diluted 1:5000 in 50 mM sodium phosphate buffer that included 0.5% BSA and 1% normal goat serum (NGS). Following 1 h incubation, the reagents that were not fixed were removed and goat anti-rabbit IgG-horseradish peroxidase compound (GAR-HRP) was added. After incubation for 30 minutes, the wells were washed and HRP substrate was added. The color reaction was stopped after 15 minutes by adding 100 µL of 1N H₂SO₄, and the absorbance measured at 450 nm with 620 nm. Each sample underwent triplicate analysis. The methodology for HMGB1 detection was the same as for HMGB2, and hence additional details are not included here. The detection limits for sTREM-1, sTREM-2, HMGB1, and HMGB2 were 93.8–6000 pg/mL, 46.9–3000 pg/mL, 1.250–80 ng/mL, and 0.625–40 ng/mL, respectively. The inter-assay coefficient of variation for all tests was <10%.

The serum HMGB1, HMGB2, sTREM-1 and sTREM-2 quartile cutoff values (25th, 50th, 75th percentiles, respectively) calculated for all participants were: HMGB1 (3.54, 4.35, 7.23 ng/mL), HMGB2 (1.25, 2.02, 5.83 ng/mL), sTREM-1 (143.54, 192.71, 272.12 pg/mL) and sTREM-2 (194.21, 329.82, 462.26 pg/mL). The cutoff values for HMGB2 in the AAA group were 1.35, 4.36, and 9.97 ng/mL, respectively.

### 2.5 Statistical Analysis

Statistical analyses were conducted using SPSS 25.0 (SPSS Inc., Chicago, IL, USA) and R software Version 4.3.2 (R Foundation for Statistical Computing, Vienna, Austria), with GraphPad Prism Version10.1.2 (GraphPad Software Inc., La Jolla, CA, USA) used for mapping. The normality test was applied using the Shapiro-Wilk method and Kolmogorov-Smirnov test. Missing values and outliers were filled with mean or median values. Normally distributed continuous variables were expressed as the mean ± standard deviation. Comparisons between the two groups were conducted using the *t*-test. Data that was not normally distributed was presented as the median (25th and 75th percentiles), and comparisons between the AAA and control groups were conducted using a non-parametric test. Categorical variables were presented as counts and percentages (n, %), and comparisons between the two groups were made using the rank-sum test. Differences across HMGB2 quartile groups were assessed using Chi-Square test for AAA incidence, and Kruskal-Wallis Test for non-normally distributed continuous variables. Pearson or Spearman correlation analysis was chosen depending on the data distribution. The relationship between various indicators and the risk of AAA was analyzed using multivariate logistic regression. To address potential bias in parameter estimation due to the small sample size (77 subjects), Firth’s penalized likelihood correction for logistic regression was performed using the logistf package in R. This method reduces bias due to small sample size, providing more reliable estimates for the association between HMGB2 levels and AAA. The receiver operating characteristic (ROC) curve and area under the ROC curve (AUC) were used to evaluate the diagnostic efficacy of HMGB1, HMGB2, and sTREM-1. For biomarkers showing inverse associations (e.g., sTREM-1), values were inverted prior to ROC analysis to ensure clinically interpretable AUC estimates. Optimal cutoff was determined via Youden Index maximization. All tests utilized a two-sided approach, with statistical significance set as *p* < 0.05.

## 3. Results

### 3.1 Basic Characteristics

A total of 77 male subjects (average age: 61.16 ± 11.00 years) were included in this study, with 36 in the AAA group (mean AAA diameter: 6.12 ± 1.72 cm) and 41 in the control group. Aneurysm rupture was reported in 4 patients in the AAA group, including 2 fatalities. No statistically significant differences were observed between the two groups in terms of age, history of former smoking, hyperlipidemia, systolic blood pressure (SBP), diastolic blood pressure (DBP), heart rate, sTREM-2, sTREM-1/sTREM-2, HDL-C, LDL-C, FBG and LVEF. Compared to the control group, the AAA group exhibited significantly higher levels of current smoking history, hypertension, HMGB1, HMGB2, TnI, NT-pro BNP, and D-Dimer (*p* < 0.05), while showing lower levels of never smoked, statin use, diabetes, sTREM-1, eGFR, TG, TC, and HbA1c (*p* < 0.05) (Table 1).

**Table 1.** **Baseline characteristics of all participants**.

	Control group (N = 41)	AAA group (N = 36)	*p*
Age (years)	59.41 ± 9.99	63.14 ± 11.89	0.144
Current smoker (n, %)	6 (14.63%)	18 (50.00%)	<0.001
Former smoker (n, %)	11 (26.83%)	5 (13.89%)	0.163
Never smoked (n, %)	24 (58.54%)	13 (36.11%)	0.049
Hypertension (n, %)	19 (46.34%)	28 (77.78%)	0.005
Hyperlipidemia (n, %)	10 (24.39%)	6 (16.67%)	0.405
Statins (n, %)	8 (19.51%)	1 (2.78%)	0.023
Diabetes (n, %)	11 (26.83%)	3 (8.33%)	0.036
Systolic blood pressure (mmHg)	130.15 ± 16.63	129.94 ± 23.25	0.966
Diastolic blood pressure (mmHg)	72.00 (65.00, 78.00)	72.00 (63.75, 85.50)	0.931
Heart rate (bpm)	78.00 (72.00, 88.00)	78.00 (68.00, 84.25)	0.292
HMGB1 (ng/mL)	4.01 (3.56, 5.02)	6.12 (3.54, 11.74)	0.020
HMGB2 (ng/mL)	1.51 (1.17, 2.60)	4.36 (1.43, 9.69)	0.002
sTREM-1 (pg/mL)	231.62 (160.80, 299.60)	176.14 (122.22, 228.87)	0.008
sTREM-2 (pg/mL)	337.28 (238.55, 485.37)	249.22 (160.72, 407.76)	0.067
sTREM-1/sTREM-2	0.63 (0.44, 0.82)	0.56 (0.40, 1.25)	0.959
eGFR (mL·min^–⁢1^·1.73 m^–⁢2^)	95.24 ± 20.15	80.61 ± 25.07	0.006
TG (mmol/L)	1.29 (0.93, 2.02)	1.06 (0.75, 1.40)	0.042
TC (mmol/L)	4.47 ± 1.00	3.93 ± 1.12	0.026
HDL-C (mmol/L)	1.09 (0.96, 1.34)	1.06 (0.91, 1.21)	0.358
LDL-C (mmol/L)	2.66 ± 0.77	2.41 ± 0.88	0.181
FBG (mmol/L)	5.48 (4.97, 5.88)	5.30 (4.88, 5.93)	0.472
HbA1c (%)	5.80 (5.50, 6.20)	5.50 (5.20, 5.90)	0.024
TnI (ng/mL)	0.01 (0.01, 0.01)	0.01 (0.01, 0.07)	<0.001
NT-pro BNP (pg/mL)	65.00 (43.50, 97.60)	140.25 (81.53, 550.58)	<0.001
D-Dimer (mg/L)	0.27 (0.19, 0.32)	1.60 (0.47, 8.86)	<0.001
LVEF (%)	68.00 (65.00, 71.00)	67.00 (57.75, 70.00)	0.089

Abbreviations: AAA, abdominal aortic aneurysm; HMGB1, high-mobility group box1; HMGB2, high-mobility group box2; sTREM-1, soluble triggering receptor expressed on myeloid cells-1; sTREM-2, soluble triggering receptor expressed on myeloid cells-2; eGFR, estimated glomerular filtration rate; TG, triglyceride; TC, total cholesterol; HDL-C, high-density lipoprotein cholesterol; LDL-C, low-density lipoprotein cholesterol; FBG, fasting blood glucose; HbA1c, glycosylated hemoglobin; TnI, troponin I; NT-pro BNP, N-terminal pro-brain natriuretic peptide; LVEF, left ventricular ejection fraction.

The overall patient cohort was divided into four groups according to HMGB2 quartiles: group A, HMGB2 <1.25 ng/mL; group B, 1.25 ≤ HMGB2 < 2.02 ng/mL; group C, 2.02 ≤ HMGB2 < 5.83 ng/mL; Group D, HMGB2 ≥5.83 ng/mL. Patients in Group D exhibited significantly greater levels of HMGB1 compared to Group A, with a notable increase in HMGB1 levels correlating with higher HMGB2 (*p* < 0.001). The prevalence of AAA in Group D was significantly higher than the other three groups, and this increased in conjunction with elevated HMGB2 levels (*p* < 0.05). The level of sTREM-1 was significantly higher in Group C compared to Groups A and B, but significantly lower in Group D (*p* < 0.001). No statistically significant differences between the groups were observed for the sTREM-2 level and the sTREM-1/sTREM-2 ratio (Table 2).

**Table 2.** **The prevalence of AAA in different serum HMGB2 quartile groups**.

	A (N = 19)	B (N = 19)	C (N = 20)	D (N = 19)	*p*
HMGB1 (ng/mL)	3.28 (2.99, 3.78)	3.82 (3.25, 4.44)*	4.97 (3.69, 6.32)*#	12.32 (9.45, 21.14)*#@	<0.001
sTREM-1 (pg/mL)	152.9 (105.18, 210.19)	177.48 (142.86, 231.62)	279.04 (217.30, 403.24)*#	181.06 (154.68, 275.66)@	<0.001
sTREM-2 (pg/mL)	302.37 (192.53, 359.17)	235.52 (152.65, 430.49)	398.22 (227.61, 903.03)*	361.58 (198.12, 463.59)	0.115
sTREM-1/sTREM-2	0.54 (0.47, 0.82)	0.75 (0.44, 1.22)	0.54 (0.38, 1.42)	0.50 (0.35, 0.76)	0.694
Prevalence of AAA (n (%))	7 (36.84)	6 (31.58)	8 (40.00)	15 (78.95)*#@	0.013

Compared to group A: *p < 0.05; compared to group B: #*p* < 0.05; compared to group C: @*p* < 0.05. Abbreviations: AAA, abdominal aortic aneurysm; HMGB1, high-mobility group box1; HMGB2, high-mobility group box2; sTREM-1, soluble triggering receptor expressed on myeloid cells-1; sTREM-2, soluble triggering receptor expressed on myeloid cells-2.

**Fig. 1.**
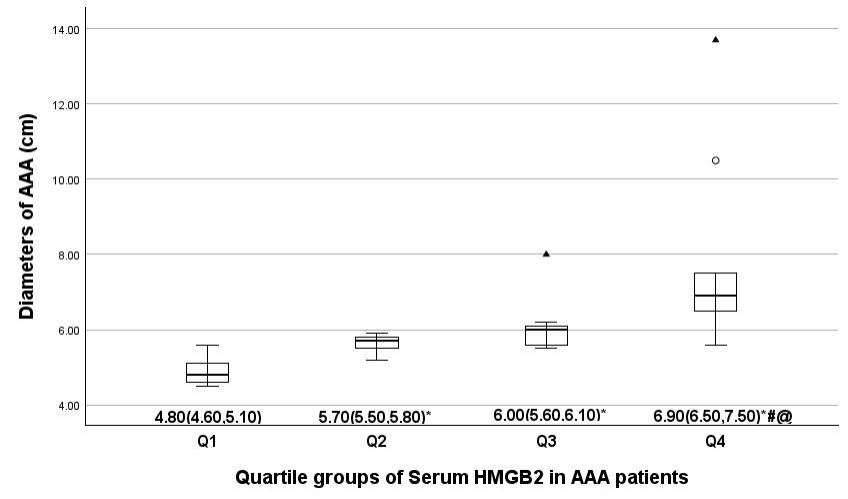
**Comparison of the AAA diameter in serum HMGB2 quartile groups**. The average AAA diameter in four serum HMGB2 quartile groups is indicated by median (25th and 75th percentiles). ▲: extreme values; ∘: outliers. Compared to group Q1: *p < 0.05; compared to group Q2: #*p* < 0.05; compared to group Q3: @*p* < 0.05. Abbreviations: AAA, abdominal aortic aneurysm; HMGB2, high-mobility group box2.

The AAA patient cohort was divided into four groups based on the HMGB2 quartiles: group Q1, HMGB2 <1.35 ng/mL; group Q2, 1.35 ≤ HMGB2 < 4.36 ng/mL; group Q3, 4.36 ≤ HMGB2 < 9.97 ng/mL; group Q4, HMGB2 ≥9.97 ng/mL. Patients in the Q4 group exhibited a significantly larger AAA diameter compared to the other three groups, with the diameter increasing significantly as the HMGB2 level increased (*p* < 0.001) (Fig. 1).

### 3.2 Correlation of Serum Biomarkers With AAA Occurrence and Clinical Parameter Interrelationships

**Fig. 2.**
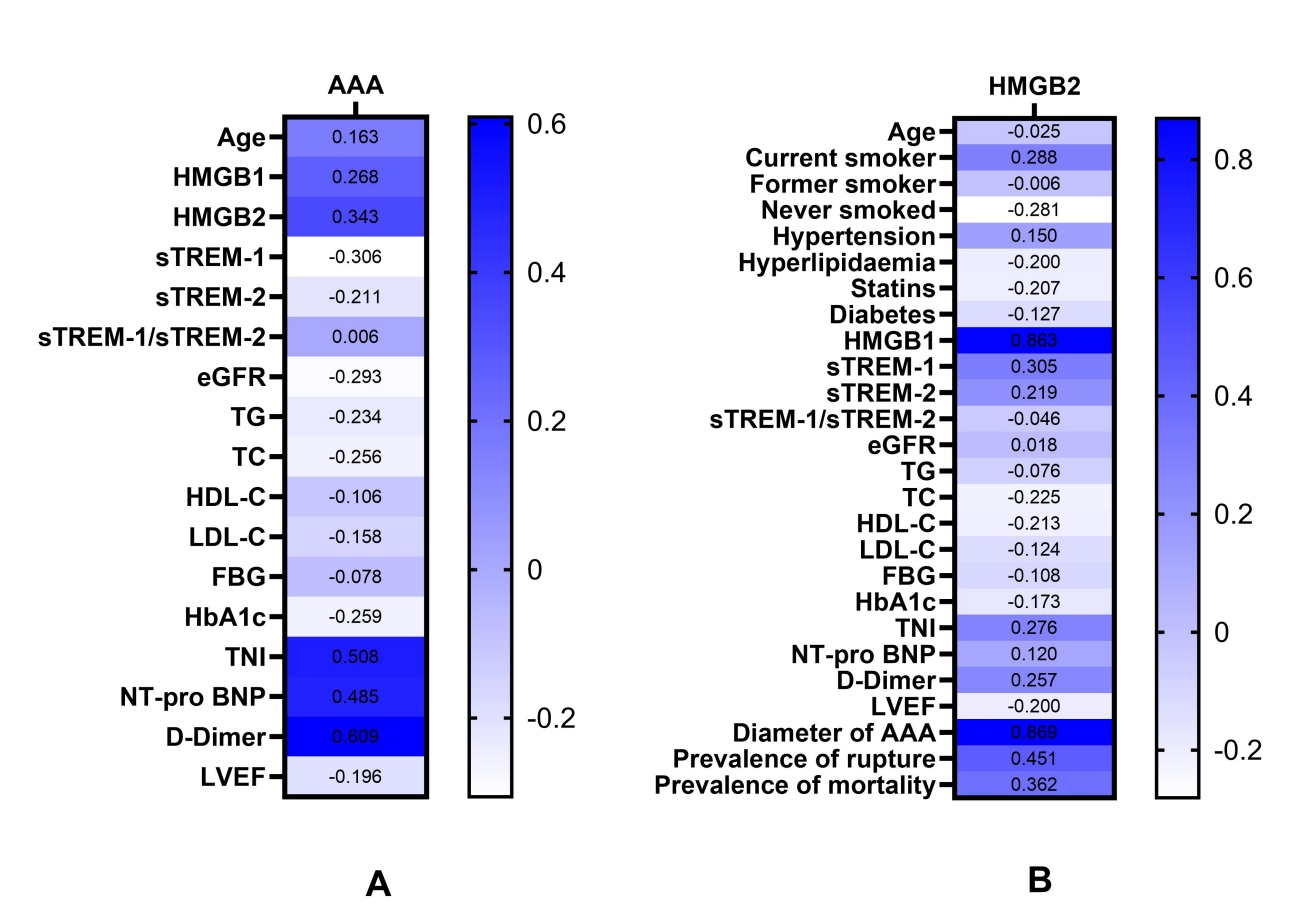
**Heat map: Correlations of various parameters with AAA and with the levels of serum HMGB2**. (A) Correlations of various parameters with the occurrence of AAA. (B) Correlations of various parameters with serum HMGB2 levels. Abbreviations: AAA, abdominal aortic aneurysm; HMGB1, high-mobility group box1; HMGB2, high-mobility group box2; sTREM-1, soluble triggering receptor expressed on myeloid cells-1; sTREM-2, soluble triggering receptor expressed on myeloid cells-2; eGFR, estimated glomerular filtration rate; TG, triglyceride; TC, total cholesterol; HDL-C, high-density lipoprotein cholesterol; LDL-C, low-density lipoprotein cholesterol; FBG, fasting blood glucose; HbA1c, glycosylated hemoglobin; TnI, troponin I; NT-pro BNP, N-terminal pro-brain natriuretic peptide; LVEF, left ventricular ejection fraction.

Spearman correlation analysis revealed that HMGB1, HMGB2, TnI, NT-pro BNP and D-Dimer were positively correlated with the occurrence of AAA (*p* < 0.05), whereas sTREM-1, eGFR, TG, TC and HbA1c were negatively correlated with AAA (Table 3 and Fig. 2).

**Table 3.** **Correlation of various parameters with AAA and serum HMGB2 level**.

Variables	AAA		HMGB2	
	r	*p*	r	*p*
Age	0.163	0.157	–0.025	0.830
Current smoker	/	/	0.288	0.011
Former smoker	/	/	–0.006	0.959
Never smoked	/	/	–0.281	0.013
Hypertension	/	/	0.150	0.194
Hyperlipidemia	/	/	–0.200	0.081
Statins	/	/	–0.207	0.070
Diabetes	/	/	–0.127	0.270
HMGB1	0.268	0.018	0.863	<0.001
HMGB2	0.343	0.002	/	/
sTREM-1	–0.306	0.007	0.305	0.007
sTREM-2	–0.211	0.066	0.219	0.055
sTREM-1/sTREM-2	0.006	0.956	–0.046	0.694
eGFR	–0.293	0.010	0.018	0.875
TG	–0.234	0.041	–0.076	0.509
TC	–0.256	0.025	–0.225	0.049
HDL-C	–0.106	0.359	–0.213	0.063
LDL-C	–0.158	0.171	–0.124	0.281
FBG	–0.078	0.578	–0.108	0.440
HbA1c	–0.259	0.023	–0.173	0.133
TnI	0.508	<0.001	0.276	0.015
NT-pro BNP	0.485	<0.001	0.120	0.301
D-Dimer	0.609	<0.001	0.257	0.026
LVEF	–0.196	0.088	–0.200	0.081
Diameter of AAA	/	/	0.869	<0.001
Prevalence of rupture	/	/	0.451	0.006
Prevalence of mortality	/	/	0.362	0.030

Abbreviations: AAA, abdominal aortic aneurysm; HMGB1, high-mobility group box1; HMGB2, high-mobility group box2; sTREM-1, soluble triggering receptor expressed on myeloid cells-1; sTREM-2, soluble triggering receptor expressed on myeloid cells-2; eGFR, estimated glomerular filtration rate; TG, triglyceride; TC, total cholesterol; HDL-C, high-density lipoprotein cholesterol; LDL-C, low-density lipoprotein cholesterol; FBG, fasting blood glucose; HbA1c, glycosylated hemoglobin; TnI, troponin I; NT-pro BNP, N-terminal pro-brain natriuretic peptide; LVEF, left ventricular ejection fraction.

Pearson or Spearman correlation analysis showed that current smoking, HMGB1, sTREM-1, TnI, D-Dimer, AAA diameter, prevalence of rupture, and mortality were positively correlated with serum HMGB2 levels, whereas never smoked and TC were negatively correlated with serum HMGB2 levels (*p* < 0.05). No significant correlations were observed between serum HMGB2 and sTREM-2 or sTREM-1/sTREM-2 (*p* > 0.05) (Table 3 and Fig. 2).

### 3.3 Association Between Serum Biomarkers and AAA

Univariate logistic regression analysis identified current smoking and hypertension as independent risk factors for AAA, while statin use and a history of diabetes were protective (*p* < 0.05). In multivariate logistic regression analysis, after adjusting for age, smoking history (including both current and former smokers), hypertension, hyperlipidemia, statin use, and diabetes (model 3), elevated levels of HMGB1 and HMGB2, and a decreased level of sTREM-1 were found to be significantly associated with increased risk of AAA (*p* < 0.05) (Table 4). To examine the predictive efficacy of different biomarkers for AAA, quartiles of serum HMGB1, HMGB2, and sTREM-1 levels were calculated and incorporated into the analysis model. All three biomarkers remained independent determinants of AAA. Compared to the first quartile, the odds ratio for AAA increased 6.925-fold (*p* = 0.045) and 8.621-fold (*p *= 0.027) in the fourth quartile of HMGB2 and HMGB1, respectively, after adjusting for the same factors as in model 3 (Table 4). To ensure the robustness of our findings, we performed Firth’s penalized likelihood regression in addition to the multivariate logistic regression analysis. The Firth-corrected estimates aligned closely with the results from multivariate logistic regression, demonstrating the robustness of the significant associations observed in the initial analysis. Additional details are provided in Table 5.

**Table 4.** **Multivariable logistic regression analysis of AAA risk factors**.

		*p*	OR	95% CI	
				Lower	Upper
Age		0.141	1.032	0.990	1.077
Current smoker		0.001	5.833	1.971	17.260
Former smoker		0.251	0.500	0.153	1.633
Never smoked		0.051	0.400	0.159	1.006
Hypertension		0.006	4.053	1.495	10.984
Hyperlipidemia		0.407	0.620	0.200	1.919
Statins		0.049	0.118	0.014	0.994
Diabetes		0.046	0.248	0.063	0.975
HMGB1					
	Model 1	0.004	1.262	1.079	1.476
	Model 2	0.005	1.248	1.070	1.455
	Model 3	0.015	1.275	1.048	1.551
HMGB2					
	Model 1	0.007	1.185	1.049	1.339
	Model 2	0.009	1.173	1.041	1.322
	Model 3	0.034	1.158	1.011	1.325
sTREM-1					
	Model 1	0.027	0.994	0.989	0.999
	Model 2	0.012	0.993	0.987	0.998
	Model 3	0.005	0.991	0.985	0.997
sTREM-2					
	Model 1	0.681	0.9998	0.9990	1.0007
	Model 2	0.455	0.9997	0.9988	1.0005
	Model 3	0.417	0.9996	0.9987	1.0005
sTREM-1/sTREM-2					
	Model 1	0.542	1.325	0.536	3.273
	Model 2	0.401	1.490	0.588	3.778
	Model 3	0.371	1.619	0.563	4.660
Quartiles of HMGB1		0.006			
	1st quartile	/	1	/	/
	2nd quartile	0.138	0.254	0.042	1.551
	3rd quartile	0.410	0.518	0.108	2.477
	4th quartile	0.027	8.621	1.278	58.145
Quartiles of HMGB2		0.040			
	1st quartile	/	1	/	/
	2nd quartile	0.829	0.828	0.150	4.563
	3rd quartile	0.560	0.609	0.115	3.232
	4th quartile	0.045	6.925	1.045	45.895
Quartiles of sTREM-1		0.008			
	1st quartile	/	1	/	/
	2nd quartile	0.621	0.643	0.112	3.698
	3rd quartile	0.313	0.398	0.067	2.377
	4th quartile	0.001	0.029	0.004	0.240

Univariable model: each smoking status was analyzed independently (current, former, never). sTREM-2: full-precision ORs and CIs for sTERM-2 are provided in 4 decimal places. Model 1: adjusted for age; Model 2: further adjusted (from Model 1) for history of smoking; Model 3: further adjusted (from Model 2) for hypertension, hyperlipidemia, statin use and diabetes. Abbreviations: AAA, abdominal aortic aneurysm; HMGB1, high-mobility group box1; HMGB2, high-mobility group box2; sTREM-1, soluble triggering receptor expressed on myeloid cells-1; sTREM-2, soluble triggering receptor expressed on myeloid cells-2.

**Table 5.** **Firth’s penalized likelihood regression analysis of AAA risk factors**.

Variable		*p*	Firth-Corrected OR	95% CI	
				Lower	Upper
Age		0.142	1.031	0.990	1.077
Current smoker		0.001	5.458	1.978	16.727
Former smoker		0.259	0.524	0.156	1.598
Never smoked		0.051	0.411	0.162	1.005
Hypertension		0.005	3.861	1.496	10.720
Hyperlipidemia		0.420	0.640	0.204	1.887
Statins		0.023	0.166	0.017	0.801
Diabetes		0.038	0.278	0.065	0.932
Model 3					
	HMGB1	0.003	1.214	1.057	1.508
	HMGB2	0.012	1.121	1.020	1.303
	sTREM-1	0.002	0.993	0.987	0.998
	sTREM-2	0.426	1.000	0.999	1.000
	sTREM-1/sTREM-2	0.377	1.556	0.573	4.212

Univariable model: each smoking status was analyzed independently (current, former, never). Model 3: all multivariable logistic regression models were tested separately for each biomarker of interest (HMGB2, HMGB1, sTREM-1, sTREM-2, and the sTREM-1/sTREM-2 ratio) in relation to the increased risk of AAA and adjusted for age, smoking history, hypertension, hyperlipidemia, statin use, and diabetes. Abbreviations: AAA, abdominal aortic aneurysm; HMGB1, high-mobility group box1; HMGB2, high-mobility group box2; sTREM-1, soluble triggering receptor expressed on myeloid cells-1; sTREM-2, soluble triggering receptor expressed on myeloid cells-2.

Following adjustment for age, smoking history, hypertension, hyperlipidemia, statin use and diabetes, the serum levels of HMGB1 (OR: 1.212, 95% CI: 1.003–1.465, *p* < 0.05) and HMGB2 (OR: 1.117, 95% CI: 1.005–1.241, *p* < 0.05) were independently associated with AAA rupture. Firth’s penalized likelihood regression was performed to address potential small-sample bias. This yielded consistent results, confirming the robust association between HMGB1, HMGB2, and AAA rupture (Table 6).

**Table 6.** **Association between HMGB1 and HMGB2 levels and AAA rupture: results from multivariable logistic regression and Firth’s penalized likelihood regression**.

Variable	*p*	OR	95% CI		*p*	Firth-Corrected OR	95% CI	
			Lower	Upper			Lower	Upper
HMGB1	0.046	1.212	1.003	1.465	0.028	1.152	1.015	1.390
HMGB2	0.041	1.117	1.005	1.241	0.030	1.089	1.008	1.207

All multivariable logistic regression models were tested separately for each biomarker of interest (HMGB2, HMGB1) in relation to the increased risk of AAA rupture and adjusted for age, smoking history, hypertension, hyperlipidemia, statin use, and diabetes. Abbreviations: AAA, abdominal aortic aneurysm; HMGB1, high-mobility group box1; HMGB2, high-mobility group box2.

### 3.4 ROC Curve Analysis of Serum HMGB2 and HMGB1 for the Prediction of AAA

**Fig. 3.**
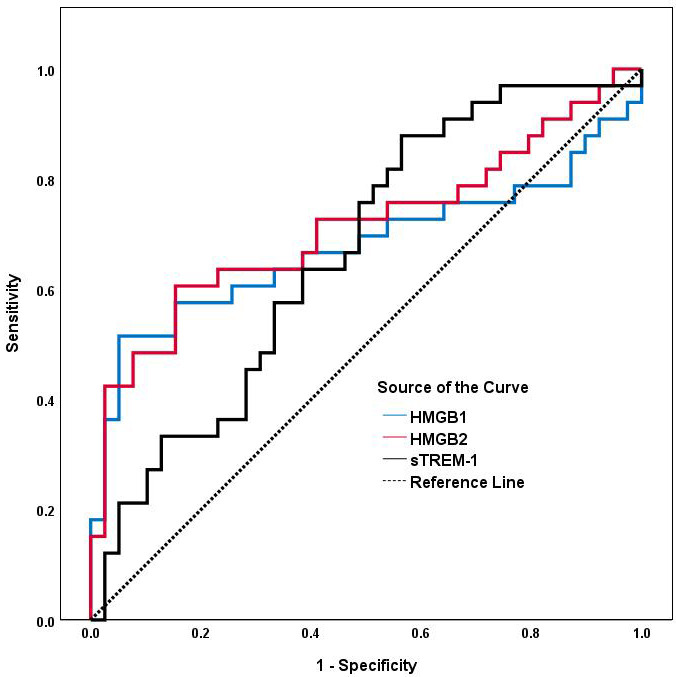
**ROC analyses of serum HMGB1, HMGB2 and sTREM-1 for predicting AAA**. The area under the ROC curve for HMGB1, HMGB2 and sTREM-1 is shown for the prediction of AAA. Abbreviations: ROC, receiver operating characteristic; AUC, area under the ROC curve; AAA, abdominal aortic aneurysm; HMGB1, high-mobility group box1; HMGB2, high-mobility group box2; sTREM-1, soluble triggering receptor expressed on myeloid cells-1.

As shown in Table 7 and Fig. 3, an HMGB2 cut-off level of 3.110 ng/mL discriminated AAA patients from controls with a sensitivity of 60.6% and specificity of 84.6% (AUC: 0.713, 95% CI: 0.588–0.839; *p* < 0.05). For HMGB1, the optimal cut-off value was 6.699 ng/mL, with a sensitivity of 51.5% and specificity of 94.9% (AUC: 0.677, 95% CI: 0.541–0.813; *p <* 0.05). For sTREM-1, the AUC value was 0.665 (95% CI: 0.540–0.790; *p* < 0.05), the optimal cut-off value was 259.289 pg/mL, with a sensitivity of 88.9% and specificity of 43.9%.

**Table 7.** **ROC curve analysis of serum HMGB2, HMGB1 and sTREM-1 for predicting AAA**.

Variable	AUC (95% CI)	*p*	Cut-off	Sensitivity	Specificity
HMGB2 (ng/mL)	0.713 (0.588–0.839)	0.001	3.110	0.606	0.846
HMGB1 (ng/mL)	0.677 (0.541–0.813)	0.011	6.699	0.515	0.949
sTREM-1 (pg/mL)	0.665 (0.540–0.790)	0.016	259.289	0.889	0.439

Abbreviations: ROC, receiver operating characteristic; AUC, area under the ROC curve; AAA, abdominal aortic aneurysm; HMGB1, high-mobility group box1; HMGB2, high-mobility group box2; sTREM-1, soluble triggering receptor expressed on myeloid cells-1.

## 4. Discussion

AAA is associated with a high mortality rate following aortic rupture and with severe effects on human health [32]. No specific biomarkers or effective drugs are currently available for the prevention, early identification and treatment of AAA [33]. Substantial evidence indicates that chronic inflammation and dysregulation of the ECM are key factors in the development of AAA, thus presenting a possible therapeutic strategy for controlling its progression [34]. Our study found that elevated serum HMGB2 and HMGB1 levels were both independently associated with the incidence and rupture of AAA in males. The Firth-corrected estimates aligned closely with the results from multiple logistic regression, demonstrating the robustness of the significant associations observed in the standard analysis. Compared to the first quartile of serum HMGB2 and HMGB1 levels, the odds ratio for AAA in the fourth quartile were increased by 6.92-fold and 8.62-fold, respectively. We also found that decreased levels of sTREM-1 were significantly associated with an increased risk of AAA. Moreover, sTREM-1 was positively correlated with serum HMGB2 levels, especially in the lower three HMGB2 quartiles, indicating that disruption of the HMGB2-TREM pathway may play a critical role in the pathogenesis of AAA.

HMGB1 is expressed in inflammatory cells, VSMCs, and endothelial cells, with previous studies also showing high abundance in human AAA lesions [13, 35, 36, 37]. Blocking HMGB1 with antibodies reduces pro-inflammatory cytokines and proteinases, and inhibits CaCl_2_-induced AAA formation [13]. The current evidence suggests that HMGB1 activation of TLR4 amplifies TLR4 signaling, thereby promoting the release of interleukin (IL) 6 and monocyte chemoattractant protein-1 (MCP-1) from VSMCs and contributing to AAA formation and progression [14]. TLRs and HMGB1 can also induce signaling cascades via RAGE [15]. The HMGB1-RAGE signaling pathway is essential for maintaining chronic inflammation during the development of AAA [16]. Our study also found that serum HMGB1 levels are independently associated with a higher risk of AAA, confirming findings from previous research. However, information on the role of HMGB2 in AAA is still limited. Previous studies have shown that it is important for both the development of atherosclerosis and coronary artery in-stent restenosis by promoting neointimal hyperplasia in mice with femoral artery injury, and for the proliferation and migration of VSMCs [19, 38]. HMGB2 exacerbates myocardial ischemic injury via reactive oxygen species (ROS)-mediated apoptosis, aberrant autophagy, and the inflammatory response [18]. Wu *et al*. [20] observed increased HMGB2 levels in an angiotensin II-induced mouse model of AAA. Moreover, these authors reported that a potential therapeutic strategy for AAA may be the inhibition of HMGB2-regulated ferroptosis and inflammation in angiotensin-II-treated VSMCs through inactivation of NF-κβ signaling. In line with previous research, we also found that elevated serum HMGB2 and HMGB1 levels were independently associated with the incidence of AAA. Our study is the first to report that serum HMGB2 levels were positively correlated with AAA diameter, with incremental increases in AAA diameter corresponding to elevated HMGB2 levels. Furthermore, we observed that higher HMGB2 levels were associated with an elevated risk of AAA rupture (OR: 1.117, 95% CI: 1.005–1.241, *p* < 0.05). Therefore, our research provides novel insights into the association between HMGB2 and AAA. HMGB2 and HMGB1 share more than 80% identity at the amino acid sequence level and possess similar biochemical properties [17]. This could account for the positive correlation observed between HMGB1 and HMGB2 levels in our study. Although HMGB1 and HMGB2 share similar structural and biochemical characteristics, they are not completely identical [39]. He *et al*. [19] demonstrated that in mice with an arterial wire injury, HMGB2 promotes neointimal hyperplasia via RAGE-mediated activation of ROS, independently of TLRs. Previous research found that HMGB2 expression in the myocardium is low under normoxic conditions, but increases 60-fold at 12 to 24 h following MI. In contrast, HMGB1 expression is moderate under normoxic conditions, and increases 3-fold after MI [18]. Furthermore, the study found that recombinant HMGB1 could dose-dependently induce HMGB2 in H9C2 cardiomyocytes. This indicates a positive feedback loop between the two HMGBs, and implies that induction of HMGB2 following MI may be partially dependent on HMGB1. ROC curve analysis in the current study found that HMGB2 and HMGB1 had diagnostic value for the detection of AAA, with good specificity but limited sensitivity. The latter may be attributed to several factors: (1) the relatively small sample size of our study, requiring larger-scale validation; (2) the complex etiology and heterogeneity of AAA, which may affect biomarker performance; (3) potential confounding influences due to population characteristics, including the severity distribution of AAA, and control group composition; (4) biological variability in biomarker expression due to various physiological and pathological factors other than AAA. While these biomarkers are promising, particularly given their high specificity, their moderate sensitivity suggests they may be more effective when used in combination with other diagnostic methods. Further large-scale investigation is required to fully elucidate the roles of HMGB2 and HMGB1 in the pathogenesis of AAA, as well as their potential application in diagnosis.

Our study found a correlation between low serum sTREM-1 levels and the occurrence of AAA, suggesting that it may offer protection against AAA. Our results contrast to those of Vandestienne *et al*. [23], who reported elevated *TREM1* mRNA expression in human aortic aneurysm tissues, and increased serum sTREM-1 levels in AAA patients. Their research indicated that TREM-1 could control angiotensin II-induced monocyte activity and promote experimental AAA. The reasons for the discrepancies between their results and the current findings are unclear, but may be attributable to several factors. First, Gibot *et al*. [40] reported that non-survivors exhibited reduced sTREM-1 levels on the first day after admission compared to survivors. sTREM-1 plasma concentrations in non-survivors remained stable or increased over time, but decreased in survivors, indicating that a high baseline sTREM-1 level is an independent protective factor in severe inflammatory conditions. Only the baseline level of sTREM-1 was assessed in our study, and hence any potential changes that occur subsequently require investigation at later follow-up times. Second, Giamarellos-Bourboulis *et al*. [41] reported that sTREM-1 functions as an anti-inflammatory mediator in sepsis, as evidenced by its positive correlations and similar kinetics with the anti-inflammatory cytokine IL-10. A decreased sTREM-1/tumor necrosis factor (TNF) ratio may promote the progression from sepsis to severe sepsis, and potentially to septic shock. Dai *et al*. [42] found that sTREM-1 plays a protective role in endothelial inflammation by inhibiting the expression of IL-1b, IL-6, TNF-α, vascular cell adhesion protein-1 (VCAM-1), and intercellular cell adhesion molecule-1 (ICAM-1) in human umbilical vein endothelial cells (HUVECs). Third, as a member of the Ig superfamily, TREM-1 is widely expressed by myeloid cells in both membrane-bound (mTREM-1) and soluble (sTREM-1) forms. sTREM-1 contains an Ig-like domain, which plays a crucial role in antigen recognition and also competes with mTREM-1 for the same ligands [43]. Consequently, sTREM-1 acts as a decoy receptor, obstructing ligands from binding to TREM-1 receptors during inflammatory responses, thereby downregulating the activation of inflammatory cytokines and exhibiting anti-inflammatory properties [44]. The protective effect of sTREM-1 in AAA patients is compromised due to its reduced levels.

Previous studies have confirmed that HMGB1 is one of the ligands for TREM-1 [25, 45, 46]. However, the relationship between HMGB2 and TREM-1 and the potential involvement of the HMGB2-TREM pathway in the pathogenesis of AAA has not been previously reported. The present findings indicate that sTREM-1 is positively correlated with HMGB2. Analysis of quartile groups based on HMGB2 levels revealed that serum sTREM-1 levels increased significantly in a concentration-dependent manner as the HMGB2 levels rose more moderately. Interestingly, a statistically significant decrease in the serum sTREM-1 level was observed in the upper quartile group of HMGB2. While sTREM-1 generally correlates positively with HMGB2, we hypothesize that there is a critical threshold beyond which this relationship inverts. Our analyses revealed a biphasic relationship between HMGB2 and sTREM-1 levels. Previous studies have also reported on the role of TREM-1 in amplifying the inflammatory response [47, 48]. We hypothesize that TREM-1 mediates the activation of its ligands, such as HMGB2, as well as inflammatory cell receptors (RAGE, TLR-4 and TLR-2), thereby initiating downstream signaling pathways that ultimately lead to AAA. The release of sTREM-1 depends on the activation and cleavage of mTREM-1 [43]. A mild to moderate increase in HMGB2 can stimulate sTREM-1 production, which acts as a decoy receptor and antagonist to mTREM-1, thus conferring anti-inflammatory properties. However, a negative feedback regulation between HMGB2 and sTREM-1 occurs when HMGB2 is highly expressed, thereby reducing its anti-inflammatory effect and contributing to the development of AAA. TREM-2 serves as a negative regulator of the inflammatory response [49]. Nonetheless, we did not observe any significant associations between sTREM-2 and AAA or HMGB2 in the current study.

Our findings indicate that current smoking and hypertension are independent risk factors for AAA, whereas the use of statins and the presence of diabetes confer protection against AAA, consistent with previous research. There is substantial evidence that smoking is a significant risk factor for AAA, with current smokers exhibiting a 5-fold increased risk and former smokers a 2-fold increase risk compared to individuals who have never smoked. Furthermore, a positive dose-response relationship was observed between the daily quantity of cigarettes smoked and the risk of developing AAA, as well as cumulative pack-years smoked and risk of AAA [50]. Although our study did not identify a significant association between hyperlipidemia and AAA, a negative correlation was observed between statin use and the incidence of AAA. Multiple Mendelian randomization analyses have implicated elevated LDL-C and reduced HDL-C levels as contributing factors to the pathogenesis of AAA [51]. Specifically, small dense LDL shows a strong association with AAA [3]. Despite these associations, there is no evidence that dyslipidemia is associated with the risk of AAA growth or rupture [52]. Several studies suggest that most statins can decrease or prevent AAA progression through various mechanisms, including regulation of endoplasmic reticulum stress, antioxidant activity, ECM synthesis, and inhibition of matrix-metalloproteinase (MMP) [53]. The increased use of statins among hyperlipidemic patients in the control group of our study may account for the observed lack of association between hyperlipidemia and AAA. Our study found a negative correlation between diabetes and AAA, consistent with results from previous research. The relationship between diabetes and a slower rate of AAA growth is well-documented [54]. However, it remains uncertain whether this effect is attributable to the diabetes itself, or to pharmacological treatments associated with its condition. In this regard, metformin, a widely used diabetes medication, has been found to mitigate matrix remodeling and inflammation in AAA [55]. The patient cohort in our study included individuals with hyperlipidemia, diabetes, or hypertension. Relevant pharmacological agents were administered during the study to treat these conditions, which may have influenced the observed outcomes.

This research has several limitations. First, it was a preliminary, retrospective, and cross-sectional study aimed at investigating the relationship between the HMGB2-TREM pathway and AAA from an inflammatory perspective. The association between the HMGB2-TREM pathway and AAA is still not fully elucidated and warrants further research. Second, we conducted a single-center, small-scale cohort study comprised of male participants only to eliminate possible sex-related confounders. The small sample size reduced the statistical power, increased the risk of Type II errors, and limited the subgroup analyses. The single-center design also introduces potential selection bias, as the study cohort may not accurately represent all populations. The exclusion of female participants, while intended to control for sex-specific confounders, prevents extrapolation of the findings to women, who exhibit distinct AAA risk profiles and pathophysiological mechanisms. In the quartile-based regression analysis, the wide confidence intervals observed for the 4th quartile of HMGB1 and HMGB2 are likely because of the small sample size of these subgroups, thus reducing the estimate precision. While point estimates suggest a strong association, the broad intervals indicate uncertainty and warrant a cautious interpretation. However, the consistent and statistically significant trend seen across quartiles supports the robustness of our findings. Due to corona virus disease 2019 (COVID-19), this study experienced difficulties with the recruitment of patients, further restricting sample diversity and potentially skewing the results toward individuals with more severe or accessible AAA cases. Additionally, only the baseline level of HMGB2 was assessed in our study. It remains to be determined whether subsequent changes in this level during follow-up could mask its role as a prognostic biomarker for the growth, rupture, or repair of AAA. Finally, our study did not investigate the mechanistic and pathological roles of the HMGB2-TREM pathway in AAA.

Given the limitations of our study, there are many issues concerning AAA that still need to be addressed in future research work. One major issue is the uncertain pathological mechanism of AAA. Further *in vitro* and *in vivo* experiments are needed to elucidate the mechanistic and pathological roles of the HMGB2-TREM pathway in AAA. These should focus on the impact of this pathway on inflammatory responses, VSMC apoptosis, and ECM degradation. Various AAA animal models and gene knockout or overexpression models could also be used to study the role of the HMGB2-TREM pathway on AAA development. The second major issue to be addressed is the difficulty of early diagnosis. To explore the potential of HMGB2 as a biomarker for the early diagnosis and prognostic assessment of AAA, dynamic changes in the serum level of HMGB2 in relation to AAA progression should be evaluated. The third issue to be addressed is the lack of effective treatment. This requires screening and development of small molecule inhibitors or agonists targeting the HMGB2-TREM pathway, followed by an evaluation of their efficacy for the prevention and treatment of AAA. Early-stage clinical trials that assess the safety and efficacy of HMGB2-TREM pathway-targeted therapies in human patients will be required. These efforts should help to identify specific biomarkers and therapeutic strategies for the early diagnosis, prognosis, and treatment of AAA patients.

## 5. Conclusion

In conclusion, elevated serum HMGB2 levels are independently associated with the incidence of AAA. Disruption of the HMGB2-TREM pathway may have a significant impact on the pathogenesis of AAA. The HMGB2-TREM pathway therefore represents a potentially novel therapeutic target for the treatment and prevention of AAA.

## Availability of Data and Materials

The data that support the findings of this study are not publicly available due to their containing information that could compromise subsequent unfinished research. The data of this study can not be disclosed until the results of the follow-up study are published.
